# Influence of Pre-reproductive Maternal Enrichment on Coping Response to Stress and Expression of c-Fos and Glucocorticoid Receptors in Adolescent Offspring

**DOI:** 10.3389/fnbeh.2017.00073

**Published:** 2017-05-09

**Authors:** Debora Cutuli, Erica Berretta, Greta Pasqualini, Paola De Bartolo, Paola Caporali, Daniela Laricchiuta, Patricia Sampedro-Piquero, Francesca Gelfo, Matteo Pesoli, Francesca Foti, Azucena Begega, Laura Petrosini

**Affiliations:** ^1^Department of Psychology, Faculty of Medicine and Psychology, Sapienza University of RomeRome, Italy; ^2^Santa Lucia FoundationRome, Italy; ^3^Department of TeCoS, Marconi UniversityRome, Italy; ^4^Department of Biological and Health Psychology, Psychology Faculty, Autonomous University of MadridMadrid, Spain; ^5^Department of Systemic Medicine, University of Rome Tor VergataRome, Italy; ^6^Department of Medical and Surgical Sciences, Magna Graecia UniversityCatanzaro, Italy; ^7^Neuroscience Laboratory, Psychology Department, University of OviedoOviedo, Spain

**Keywords:** environmental enrichment, rats, maternal behavior, coping, glucocorticoids, brain activation

## Abstract

Environmental enrichment (EE) is an experimental setting broadly used for investigating the effects of complex social, cognitive, and sensorimotor stimulations on brain structure and function. Recent studies point out that parental EE experience, even occurring in the pre-reproductive phase, affects neural development and behavioral trajectories of the offspring. In the present study we investigated the influences of pre-reproductive EE of female rats on maternal behavior and adolescent male offspring's coping response to an inescapable stressful situation after chronic social isolation. For this purpose female Wistar rats were housed from weaning to breeding age in enriched or standard environments. Subsequently, all females were mated and housed in standard conditions until offspring weaning. On the first *post partum* day (ppd 1), mother-pup interactions in undisturbed conditions were recorded. Further, after weaning the male pups were reared for 2 weeks under social isolation or in standard conditions, and then submitted or not to a single-session Forced Swim Test (FST). Offspring's neuronal activation and plastic changes were identified by immunohistochemistry for c-Fos and glucocorticoid receptors (GRs), and assessed by using stereological analysis. The biochemical correlates were measured in the hippocampus, amygdala and cingulate cortex, structures involved in hypothalamic-pituitary-adrenocortical axis regulation. Enriched dams exhibited increased Crouching levels in comparison to standard reared dams. In the offspring of both kinds of dams, social isolation reduced body weight, decreased Immobility, and increased Swimming during FST. Moreover, isolated offspring of enriched dams exhibited higher levels of Climbing in comparison to controls. Interestingly, in the amygdala of both isolated and control offspring of enriched dams we found a lower number of c-Fos immunopositive cells in response to FST and a higher number of GRs in comparison to the offspring of standard dams. These results highlight the profound influence of a stressful condition, such as the social isolation, on the brain of adolescent rats, and underline intergenerational effects of maternal experiences in regulating the offspring response to stress.

## Introduction

Environmental enrichment (EE) is an experimental setting broadly used for investigating the effects of potentiating social interactions as well as increasing the exposure to sensorimotor and cognitive stimulations on brain structure and function (Rosenzweig et al., 1964; Nithianantharajah and Hannan, 2006, 2009). EE exerts beneficial effects on many behavioral (improved motor and cognitive performances, reduced reactivity to stress), morphological (increased dendritic arborization, spine number, synaptic density, and neurogenesis), and molecular (modifications in gene expression, neurotrophic factors, and neurotransmission) brain features (Nithianantharajah and Hannan, 2006, 2009; Petrosini et al., 2009; Baroncelli et al., 2010; Simpson and Kelly, 2011; Sale et al., 2014).

In spite of the vast literature on the positive effects of EE when applied immediately after weaning, in adulthood, during aging or even in the presence of brain damage, the transgenerational beneficial effects of pre-reproductive EE have been only recently examined (Arai et al., 2009; Arai and Feig, 2011; Leshem and Schulkin, 2012; Mashoodh et al., 2012; Caporali et al., 2014, 2015; Cutuli et al., 2015). And yet, this issue is noteworthy given that parental environmental experience may imprint offspring's phenotype over generations through many epigenetic processes (Weaver, 2007; Kanherkar et al., 2014).

Interactions between individual and environment take place lifelong from conception. In fact, during fetal development besides the overwhelming impact of the genetic control, even the environmental stimuli strongly influence the highly susceptible developing structures. Namely, the environment experienced by the pregnant mother exerts substantial effects on the intrauterine milieu and affects fetal organogenesis. During pregnancy, the maternal exposure either to negative (radiations, contaminants, alcohol, drugs, prenatal stress, food deprivation, or micronutrient deficiency; Rice and Barone, 2000; Meck and Williams, 2003; Van den Bergh et al., 2005; Weinstock, 2005; Mueller and Bale, 2008; Swanson et al., 2009; Thompson et al., 2009; Charil et al., 2010; Glover, 2011) or positive (complex environments, voluntary exercise, dietary supplementation; McKim and Thompson, 1975; Kiyono et al., 1985; Dell and Rose, 1987; Parnpiansil et al., 2003; Bick-Sander et al., 2006; Lee et al., 2006; Welberg et al., 2006; Sale et al., 2007; Herring et al., 2012; Leshem and Schulkin, 2012; Mychasiuk et al., 2012; Rosenfeld and Weller, 2012) environmental stimulations may exert deleterious or beneficial impact, respectively, on physical and behavioral offspring's development.

In previous studies (Caporali et al., 2014, 2015; Cutuli et al., 2015) we demonstrated the beneficial influences of pre-reproductive EE on maternal care and offspring's motor and cognitive performances as well as on neurotrophic functioning.

On such a basis the current research was aimed to analyze whether and how pre-reproductive EE of female rats may influence even the effects of a chronic stress, such as social isolation, on their adolescent offspring. In fact, it is accepted that social isolation at adolescence represents a valid tool for understanding the impact of social stress given that adolescent rodents live in groups and exhibit high levels of social behavior (Panksepp et al., 2007). To this aim glucocorticoid receptors (GRs) expression in cortico-limbic networks was assessed as a marker of stress adaptation in the offspring of pre-reproductively enriched or standard dams. It is well-known that glucocorticoids are the end product of hypothalamic-pituitary-adrenal (HPA) axis activation and have large effects on adaptive behaviors and pathophysiology of several stress-related disorders, such as depression, anxiety, and drug abuse (Holsboer, 2000; McEwen, 2000; Sapolsky, 2000; Roozendaal and McGaugh, 2011; Lee and Sawa, 2014). Furthermore, we evaluated the interactions of pre-reproductive maternal EE and chronic social isolation on the coping response of the adolescent offspring to an inescapable stressful situation (Forced Swim Test, FST), and on cortico-limbic network activation (c-Fos). Indeed, hippocampus, amygdala, and cingulate cortex, are closely involved in HPA axis regulation (Sapolsky, 2003; Sarrazin et al., 2009; Nicolaides et al., 2015). To gain further indications about the possible mechanisms through which pre-reproductive maternal rearing conditions may influence the offspring's phenotype, we also assessed the early maternal care of pre-reproductively enriched or standard reared lactating dams on the first *post partum* day (ppd 1).

## Materials and methods

### Experimental design

#### Maternal housing conditions

Twenty female 21-day old Wistar rats were randomly assigned to enriched or standard rearing conditions.

From post natal day (pnd) 21 to 72 Enriched Females (EF) were housed in a group of 10 in a large cage (100 × 70 × 90 cm) as described previous works (Cutuli et al., 2011, 2015; Caporali et al., 2014, 2015). During the enrichment period the toys and objects inside the enrichment cage were changed twice a week, while the feeding boxes and water bottles were moved to different cage areas once a week in order to promote explorative behaviors. Moreover, each enriched animal was handled 10 min daily.

Standard Females (SF) were pair-housed in standard cages (40 × 26 × 18 cm) containing wood sawdust, a red plastic tube and no toys. Food and water were delivered *ad libitum* through dispensers kept always in the same position. SF received the usual care by the animal house staff without any extra-manipulations. This procedure avoided an impoverished rearing and allowed being accustomed to the human contact.

A 12/12 h dark/light cycle (light on between 07:00 a.m. and 07:00 p.m.) was applied to both EF and SF groups. On the pnd 72, the animals were weighted and EF were pair-housed in standard cages to be accustomed to the novel rearing condition. After a week, each EF and SF specimen in oestrus phase (Marcondes et al., 2002; Sayin et al., 2014) was caged for 5 days with a standard-reared male rat (≈300 g) to allow mating. Afterwards, male rats were removed, and the females were maintained in standard home cages throughout pregnancy, delivery and until offspring's weaning. At this stage females were again weighted and maintained in standard cages to be used for other experiments.

All efforts were made to minimize animal suffering and reduce the number of animals that were used, per the European Directive (2010/63/EU). All procedures were approved by the Italian Ministry of Health.

#### Experimental groups of pups

At birth (pnd 0), culling of the litters was quickly performed and pups' weight was recorded (all procedures taking max 5 min) reducing the litters to five males and five females. Litters not compliant with the condition were excluded from analyses. Litters from eight EF and eight SF dams were used. Depending on the maternal rearing conditions, two groups of male pups were obtained: one group encompassed the pups of EF (*n* = 24); the other group encompassed the pups of SF (*n* = 24). Notably, the difference in housing conditions affected the mothers in their pre-reproductive life and not the pups, which were reared in standard conditions.

After weaning, EF and SF offspring were further divided in two groups: standard reared or socially isolated groups. Standard rearing consisted in being pair-housed in standard cages containing wood sawdust, a red plastic tube and no toys. The social isolation lasted 2 weeks and consisted in being individually-housed in standard conditions. In both settings food and water were delivered *ad libitum* through dispensers kept always in the same position. Both groups received the usual care by the animal house staff without any extra-manipulations.

Four groups (*n* = 12/group) of adolescent animals were then obtained:

- Pups from EF reared in standard conditions (group name: EF-p);
- Pups from SF reared in standard conditions (group name: SF-p);
- Isolated pups from EF (group name: EF-p iso);
- Isolated pups from SF (group name: SF-p iso).

**Figure 1:**
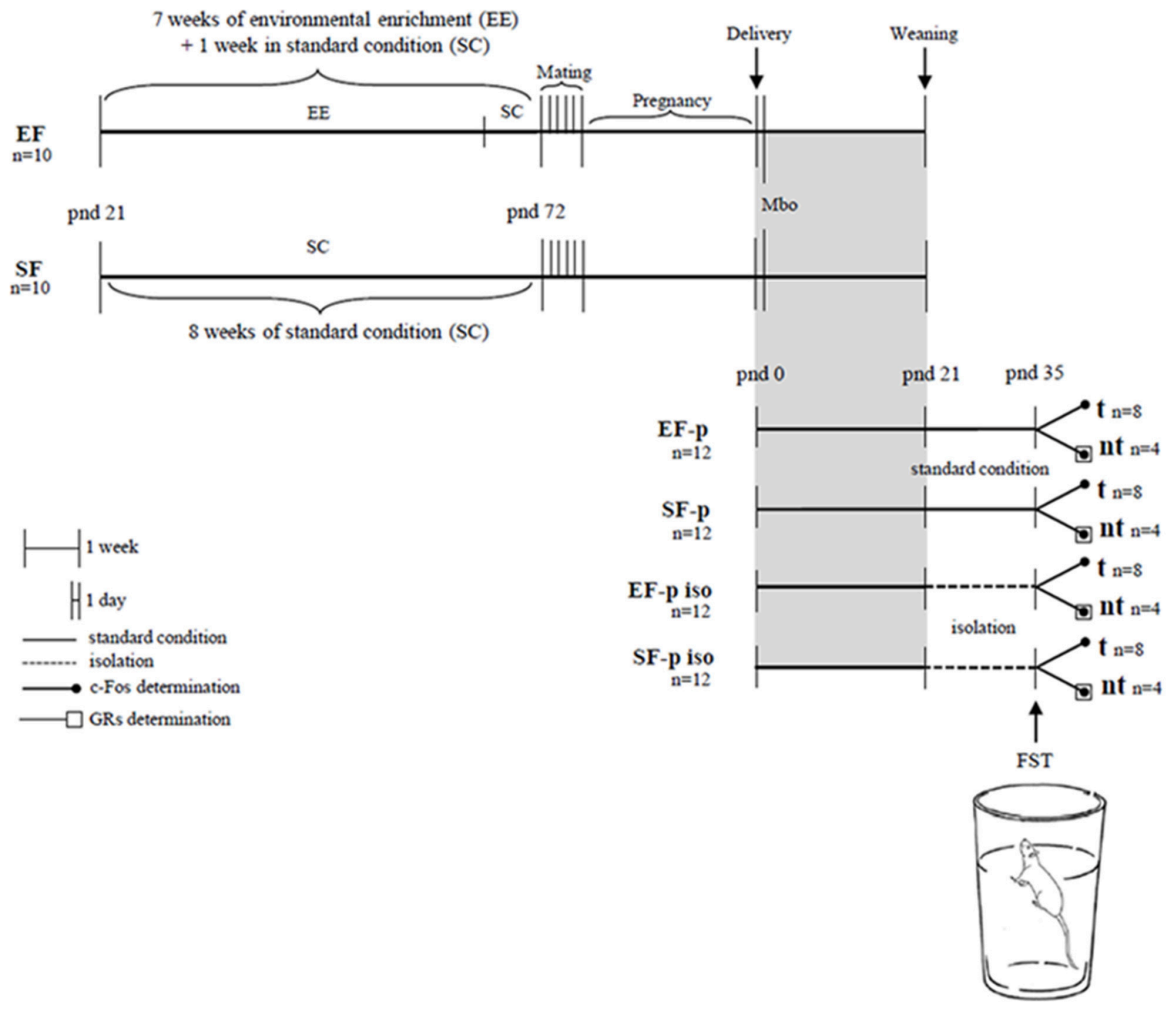
**Global timing of the experimental design**. Experimental groups of female rats according to different pre-reproductive housing conditions (EF, Enriched Females; SF, Standard Females). Experimental groups of male pups (EF-p, pups from EF reared in standard conditions; SF-p, pups from SF reared in standard conditions; EF-p iso, isolated pups from EF; SF-p iso, isolated pups from SF; t, tested pups; nt, no tested pups), behavioral testing (Mbo, Maternal behavior observations; FST, Forced Swim Test), and biochemical analyses (c-Fos and GRs determination).

At pnd 35 pups' body weight was collected. One pup per dam (*n* = 8/group) was behaviorally evaluated in the FST. For c-Fos quantification we used 4 animals randomly selected from FST tested rats (tested group, t) and 4 not behaviorally tested siblings of the four remaining t pups (no tested group, nt). This analysis was performed to evaluate whether the cortico-limbic neuronal activation in response to an acute stressful situation (FST) was modulated by maternal enrichment and/or social isolation. To assess whether these two variables induced long-term plastic changes in the glucocorticoid system, GRs levels were analyzed in the nt pups (*n* = 4/group). Our experimental protocol allowed a sampling in which in all groups each animal belonged to a different litter. Global timing of experimental procedures is reported in Figure 1.

### Behavioral testing

#### Maternal behavior observations

Maternal behavior in rats consists of “a constellation of preparatory and caretaking behaviors” (Kristal, 2009) that ensures pups' survival and promotes offspring's development.

To assess maternal behavior in basal conditions, at ppd 1 mother-pups interactions of EF and SF were recorded for four 20-min observation periods (09:00 a.m., 12:30 a.m., 04:00 p.m., 07:30 p.m.). Observations collected during the dark phase of the light/dark cycle were performed under dim red light illumination. Within each observation period the following behaviors of mothers were scored for duration and frequency (Fleming and Rosenblatt, 1974; Petruzzi et al., 1995; Venerosi et al., 2008; Cutuli et al., 2015):

- *Pup-directed Behaviors*:
- Retrieving: the dam was picking up any pup in her mouth and carrying it to the nest;
- Licking: the dam was licking or grooming any part of the pup's body, primarily the anogenital region;
- Sniffing: the dam was sniffing one or more pups;
- Nursing: part of the litter was attached to dam's nipples while the dam did not show obvious back-arching;
- Crouching (or arched-back nursing): the dam laid over all pups with the body arched, hind-limbs splayed, and no apparent movement;
- Nest Building: the dam was pushing and pulling the sawdust or the plastic tube toward the pups to form or adjust the nest.
- *Non-pup-directed behaviors*:
- Digging: the dam was nuzzling in the sawdust out of nest area, pushing and kicking it around using the snout and/or both fore- and hind-paws;
- Grooming: the dam was wiping, licking, combing or scratching any part of its own body;
- Wall Rearing: the dam was rearing on hindlimbs, while leaning (or not) with the forelimbs on the cage walls, often sniffing the air;
- Exploring: the dam was moving around the cage and sniffing the substrate, but not carrying pups or nesting material;
- Resting: the dam was lying down alone, out of the nest;
- Drink or Eat: the dam was drinking or eating, out of the nest.
- *Other behaviors:* all behaviors different from the ones classified in the previous categories.

Manual scoring of the maternal behavior was performed by a researcher blind to pre-reproductive rearing condition of the dams, by using EthoVision XT (Noldus).

#### Forced swim test (FST)

The FST is a well-validated test based on the rodent's response to the threat of drowning (Porsolt et al., 1978). Although FST is usually performed in two sessions (24 h or longer apart) in order to measure susceptibility to negative mood, we exposed the animals to a single testing session because our aim was to evaluate their coping strategies in a stressful condition (Andolina et al., 2013, 2015; Cutuli et al., 2016). At pnd 35 each adolescent rat of t groups was gently placed in a glass cylinder (height 45.5 cm, diameter 18 cm) containing 20 cm of water at 28 ± 2°C for 5 min (Bernal-Morales et al., 2009; Kokras et al., 2009; Rosenfeld and Weller, 2012; Karimi et al., 2014; de Kloet and Molendijk, 2016). After testing, rats were removed from the cylinder, dried with absorbent paper, and put back in the homecages. The cylinder was cleaned and water was changed between tests.

The behavior of each animal during FST was recorded by using a frontally-mounted camera. An observer blind to the animal's grouping manually scored the videos (EthoVision XT, Noldus). Duration of the following behavioral items was measured:

- Immobility: total absence of active movements besides minor efforts to keep the head afloat;
- Swimming: active swimming with the animal pedaling and moving around the cylinder with all four paws immersed under water;
- Climbing: vigorous attempts to climb the walls of the cylinder with the animal floating upright extending their front paws.

According to Costa et al. (2013) and Keers et al. (2012), Immobility was considered as a passive coping strategy, while Climbing and Swimming were considered as active coping strategies.

### Biochemical analyses

#### Tissue preparation

Before perfusion, t rats (at the end of FST) and nt rats were isolated for 1 h to perform c-Fos immunohistochemistry (modified from Passino et al., 2002).

**Figure 2:**
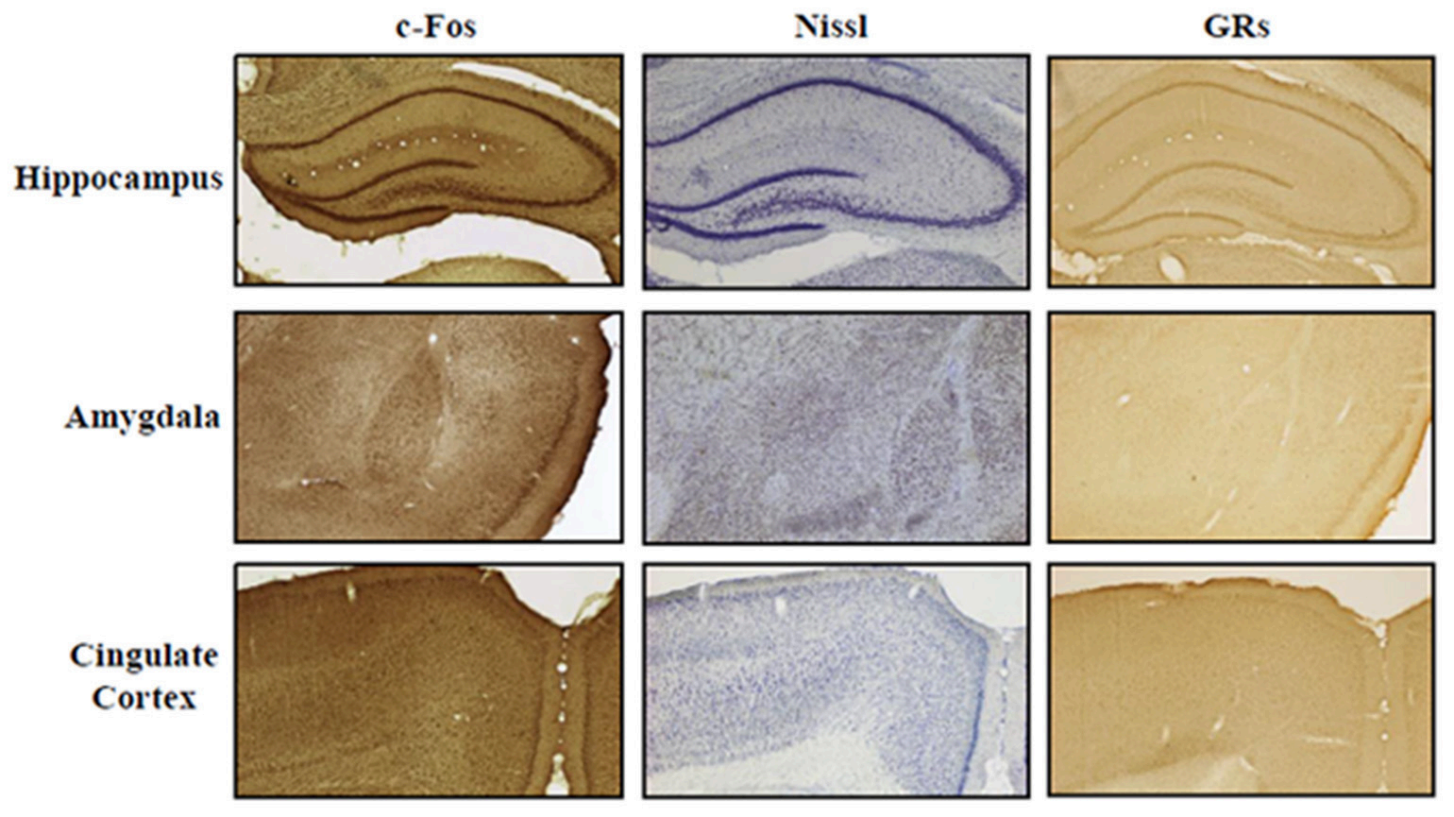
**ROI representation**. Representative photomicrographs (4× magnification) of Hippocampus, Amygdala, and Cingulate Cortex. Staining: c-Fos, Nissl, and glucocorticoid receptors (GRs). Scale bar: 500 μm.

Rats were deeply anesthetized and transcardially perfused with saline (0.9% NaCl) followed by 4% paraformaldehyde fixative in 0.1 M phosphate buffer (pH 7.4). The brains were removed and cryoprotected with 30% sucrose in phosphate buffer. Anterior brains were cut in serial coronal sections (40 μm thickness) with a freezing microtome and alternatively processed for Nissl staining, c-Fos and GRs immunohistochemistry. According to the rat stereotaxic atlas (Paxinos and Watson, 1998) using Nissl sections as anatomical reference, the following regions of interest (ROI) were identified: hippocampus (Hp, from −1.60 to −6.80 mm in relation to bregma), amygdala (Amyg, from −1.60 to −4.80), cingulate cortex (Cg, from +3.70 to −1.40; Figure 2).

#### c-Fos immunohistochemistry

Free floating coronal sections were washed three times with Phosphate-Buffered Saline (PBS, pH 7.4) and then incubated for 30 min in 0.3% H_2_O_2_ in PBS in order to prevent endogenous peroxidase. After three washes in PBS, the sections were incubated for 2 h in blocking solution (BS): PBS-T (0.25% Triton X-100) containing 3% Normal Goat Serum (Vector Laboratories). All steps were performed at room temperature. Sections were incubated for 48 h at 4°C with primary antibody (1:300, Rabbit polyclonal anti-c-Fos, ab7963, Abcam) in BS.

After washing six times with PBS, the sections were incubated for 2 h in BS containing secondary antibody (1:200, biotinylated Goat anti-Rabbit, Vectastain Elite ABC Kit, Vector Laboratories). After three washes in PBS, the sections were incubated for 1 h in Avidin-Biotin complex (Vectastain Elite ABC Kit, Vector Laboratories) in PBS-T (0.5% Triton X-100). Sections were washed three times in PBS and then visualized with diaminobenzidine, as chromogen (DAB, ScyTek Laboratories). Finally, the sections were washed three times in PBS, dehydrated in ethanol, cleared in xylene and coverslipped with Eukitt (O. Kindler GmbH). All steps were performed at room temperature. We confirmed the specificity of the immunohistochemical pattern by omitting the primary antibody. This negative control resulted in the absence of c-Fos immunoreactivity in the investigated brain regions.

To assess differences between experimental groups, the number of c-Fos immunopositive (^+^) cells of t rats of each group was compared to the number of c-Fos^+^ cells of the respective nt rats. A c-Fos activation index was also calculated by using weighted Δ values: number of c-Fos^+^ cells in each subject exposed to FST *minus* mean number of c-Fos^+^ cells of nt respective group (Δ)/number of c-Fos^+^ cells in each subject exposed to FST *plus* mean number of c-Fos^+^ cells of nt respective group.

#### GRs immunohistochemistry

GRs immunohistochemistry was performed according to a modified version of the protocol provided by Sampedro-Piquero et al. (2014). Free-floating coronal sections were washed three times with Tris-Buffered Saline (TBS, pH 7.4)-T (0.1% Triton X-100) and then incubated for 30 min in 3% H_2_O_2_ in TBS in order to prevent endogenous peroxidase. After three washes in TBS-T sections were incubated for 2 h in blocking solution (BS): TBS-T containing 3% Normal Goat Serum (Vector Laboratories) and then washed three times in TBS-T. All steps were performed at room temperature. Sections were incubated for 48 h at 4°C with primary antibody (1:400, GR polyclonal Rabbit antibody, M-20, Santa Cruz Biotechnology) in BS. After washing three times with TBS-T sections were incubated for 2 h in TBS containing 3% Normal Goat Serum and secondary antibody (1:200, biotinylated Goat anti-Rabbit, Vectastain Elite ABC Kit, Vector Laboratories). After three washes in TBS, the sections were incubated for 1 h in Avidin-Biotin complex (Vectastain Elite ABC Kit, Vector Laboratories) in TBS.

The sections were washed three times in TBS and then visualized with diaminobenzidine, as chromogen (DAB, ScyTek Laboratories). Finally, sections were washed three times in TBS and dehydrated in ethanol, cleared in xylene and coverslipped with Eukitt (O. Kindler GmbH). All steps were performed at room temperature. We confirmed the specificity of the immunohistochemical pattern by omitting the primary antibody. This negative control resulted in the absence of GRs immunoreactivity in the investigated brain regions.

#### Cell counting and stereological quantification

Stained sections were analyzed by using a light microscope (Axioskop 2, Zeiss). Stereological quantification of c-Fos^+^ and GRs^+^ cells was performed online by using the Stereo Investigator software (mbf bioscience; MicroBrightField). ROI were outlined using a 4x objective lens, cell counting was performed using Optical Fractionator probe at a higher magnification (100x oil-immersion objective lens). The stereological analysis was carried out on 40 μm coronal slides with a 9 sampling interval between sections. A 175 × 112 μm grid with a 20 × 20 μm counting frame was systematically and randomly superimposed on ROI. The Optical Disector height was 20 μm with a 4 μm guard zone. Cell counting was performed on three sections for each ROI. The measure used for calculating the Optical Fractionator results was the “Estimated cell population using mean section thickness with counts.” These Optical Fractionator results are based on thick sections and estimate the total cell population number in a volume on the basis of the number of cells sampled with a systematic random sampling set of unbiased virtual counting spaces covering the entire ROI. This is accomplished by systematically sampling a known fraction of the section thickness, a known fraction of sectional area and a known fraction of the sections that contain the ROI. It provides an unbiased cell quantification, unaffected by tissue shrinkage. Parameters of stereological analysis are detailed in Table 1.

**Table 1:** **Parameters used for stereological analyses of c-Fos^+^ and GRs^+^ neurons in bilateral Hippocampus, Amygdala, and Cingulate Cortex**.

Section cut thickness	40 μm
Section evaluation interval	9
Tracking method	Simple click
Counting frame size	20 × 20 μm
SRS grid size	175 × 112 μm (x − y)
Percentage of sampled region of interest	5%
Optical disector top/bottom guard zone	4 μm
Optical disector height	20 μm
Coefficient of error (CE)	Gundersen (*m* = 1)
Optical fractionator estimating cell population	Mean section thickness with counts

### Statistical analysis

Statistical analyses were performed by using STATISTICA 7.0 (StatSoft). Data were firstly tested for normality (Wilk-Shapiro's test) and homoscedasticity (Levene's test). Since behavioral and biochemical data did not fully meet parametric assumptions, non-parametric analyses of variance (Kruskal–Wallis test, Mann–Whitney U) were used.

Dams' and pups' body weight was analyzed by one- and two-way ANOVAs for independent measures (maternal rearing condition, social isolation). Body weight of all pups from one litter was averaged and counted as a single biological sample to maintain the dam as the experimental unit. Differences were considered significant at the *p* < 0.05 level.

## Results

### Dams' and pups' body weight

At end of the EE exposure (pnd 72), EF weight was significantly inferior (191.91 ± 2.56 g) to the one of SF [230.35 ± 9.74 g; One-way ANOVA: *F*_(1, 14)_ = 14.56, *p* = 0.002], while no difference in maternal weight was observed at offspring's weaning [EF: 277.50 ± 3.75 g, SF: 292.45 ± 12.55 g; One-way ANOVA: *F*_(1, 14)_ = 1.30, *p* = 0.27].

**Figure 3:**
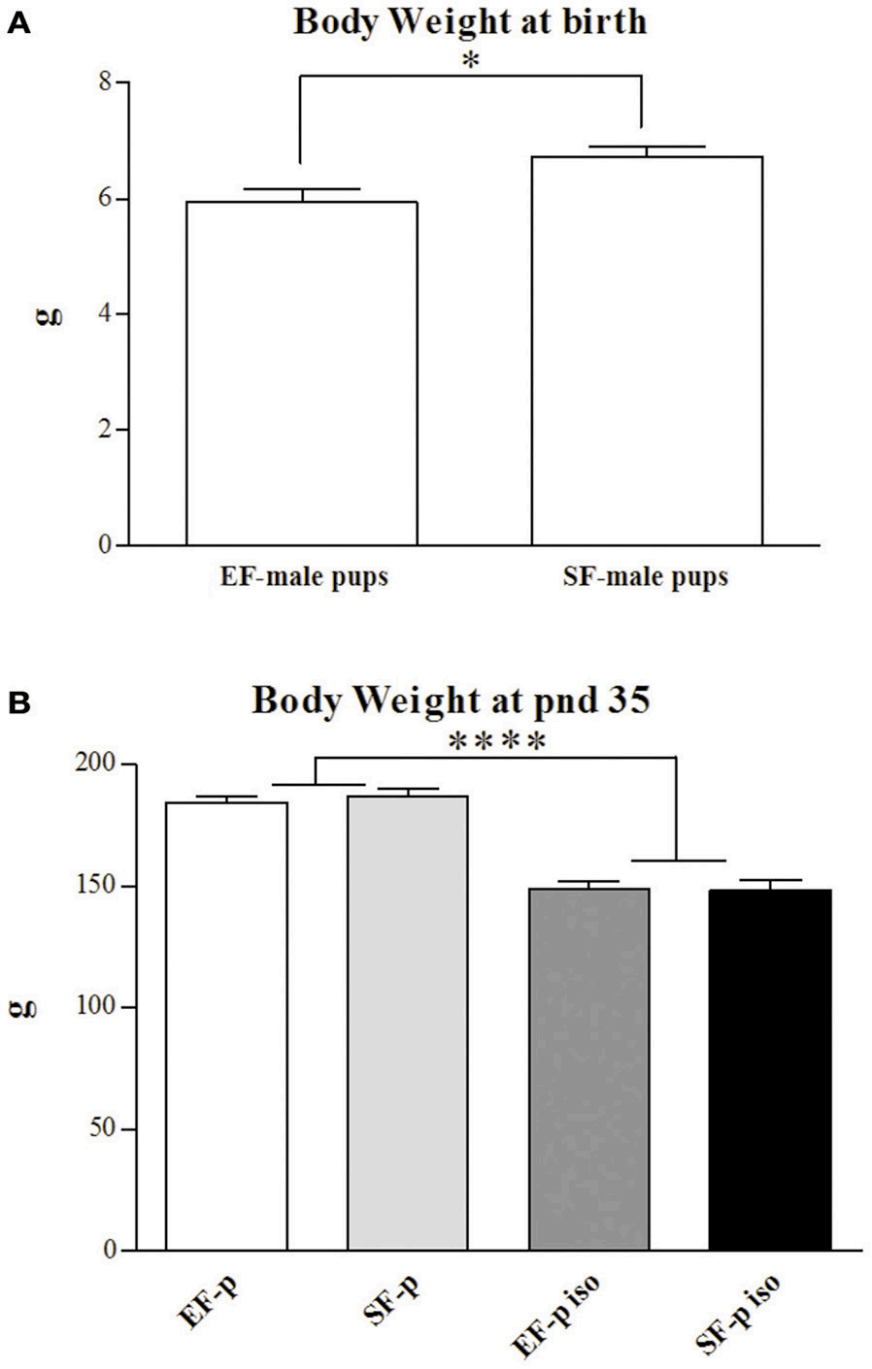
**Body weight**. Histograms show animals body weight (g) measured at birth in EF- and SF-male pups **(A)** and at pnd 35 in EF-p, SF-p, EF-p iso, SF-p iso groups **(B)**. All data are presented as mean ± SEM. Asterisks inside the graphs indicate the significance of comparisons between groups: ^*^p < 0.05, ^****^p < 0.000001.

At birth, EF male pups' weight was significantly lower than SF male pups' one [One-way ANOVA: *F*_(1, 14)_ = 7.14, *p* = 0.02; Figure 3A]. A two-way ANOVA (maternal rearing condition × social isolation) performed on offspring's body weight at adolescence revealed a significant effect of social isolation [*F*_(1, 28)_ = 138.96, *p* < 0.000001], while maternal rearing condition [*F*_(1, 28)_ = 0.12, *p* = 0.73] and interaction [*F*_(1, 28)_ = 0.29, *p* = 0.59] were not significant (Figure 3B).

Thus, isolated pups exhibited a marked loss of weight regardless of maternal pre-reproductive experience.

### Maternal behavior observations

**Figure 4:**
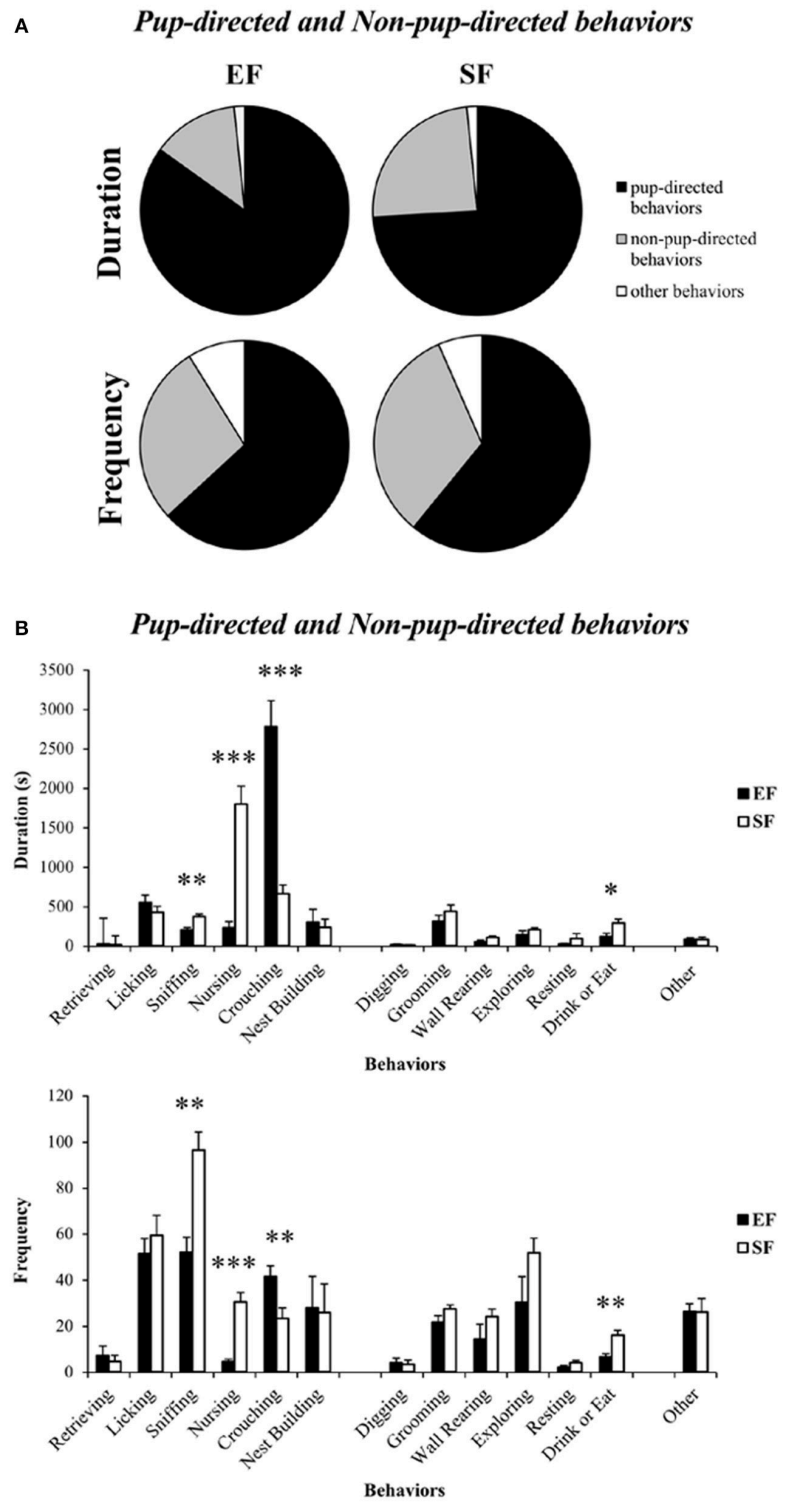
**Maternal behavior observations**. Pie charts **(A)** and histograms **(B)** show duration (s) and frequency of *Pup-directed, Non-pup-directed* and *Other behaviors* in EF and SF dams. ^*^p < 0.05, ^**^p < 0.01, ^***^p < 0.001.

Non-parametric analyses (Mann–Whitney U) on duration and frequency of the sum of *Pup-directed* vs. *Non-pup-directed behaviors* at ppd 1 revealed significantly increased duration of the *Pup-directed behaviors* accompanied by significantly decreased duration and tendentially decreased frequency of *Non-pup-directed behaviors* in EF dams in comparison to the SF dams (Figure 4A; see Table S1). No differences between EF and SF groups were observed in duration of *Other behaviors* as well as in frequency of *Pup-directed* and *Other behaviors* (see Table S1).

Detailed analyses on single *Pup-directed behaviors* demonstrated that EF dams exhibited more Crouching and less Sniffing and Nursing than SF dams (Figure 3B; see Table S1). As for *Non-pup-directed behaviors*, EF dams emitted less Drink or Eat and tendentially less Wall Rearing than SF dams (Figure 4B; see Table S1).

### Forced swim test (FST)

Non-parametric analyses (Kruskal–Wallis tests) performed among the four groups on Immobility (*H* = 13.06, *p* = 0.004), Swimming (*H* = 11.89, *p* = 0.008), and Climbing (*H* = 10.57, *p* = 0.01) were significant.

**Figure 5:**
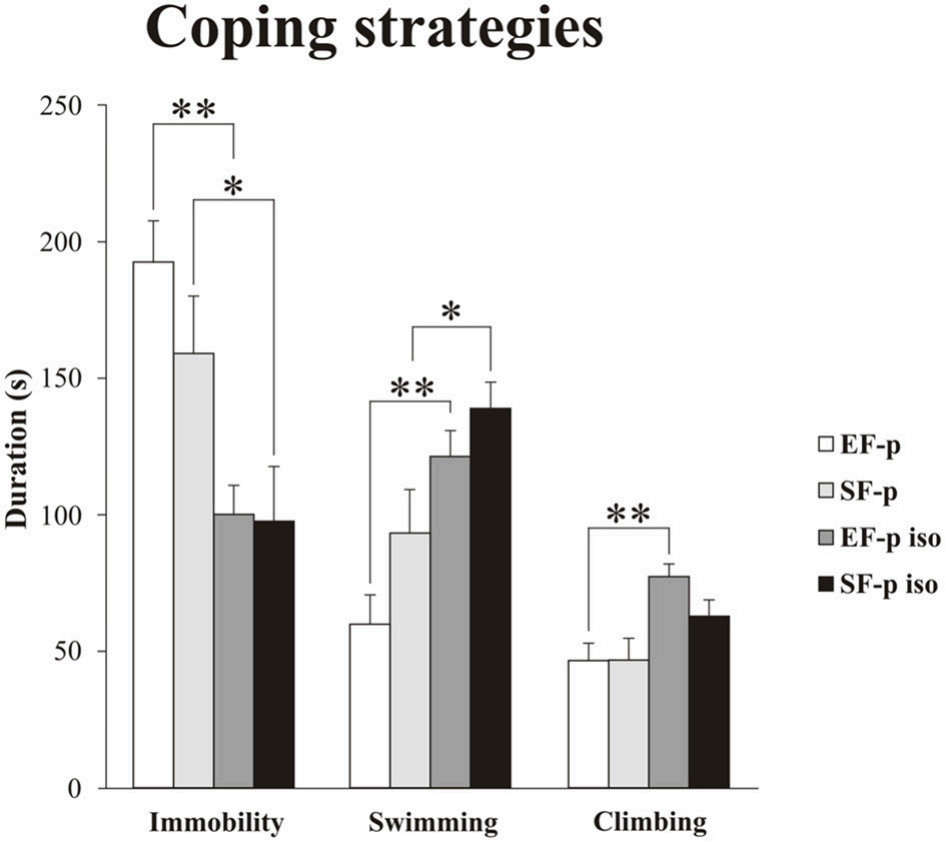
**Coping strategies in Forced Swim Test (FST)**. Histograms show duration (s) of behavioral parameters (Immobility, Swimming, Climbing) in EF-p, SF-p, EF-p iso, SF-p iso rats subjected to FST (t). ^*^p < 0.05, ^**^p < 0.01.

As for the passive coping strategy, analyses between groups (Mann–Whitney U) demonstrated that social isolation was able to reduce duration of Immobility (EF-p iso *vs*. EF-p: *U* = 2, *p* = 0.002; SF-p iso vs. SF-p: *U* = 13, *p* = 0.046) in both isolated groups (EF-p iso and SF-p iso; Figure 5). Comparisons between Immobility levels of EF-p vs. SF-p (*U* = 24, *p* = 0.40) and EF-p iso vs. SF-p iso (*U* = 28, *p* = 0.67) were not significant.

As for active coping strategies, analyses between groups (Mann-Whitney *U*) demonstrated that social isolation similarly increased Swimming in both EF-p iso and SF-p iso (EF-p iso vs. EF-p: *U* = 4, *p* = 0.003; SF-p iso vs. SF-p: *U* = 12, *p* = 0.03), while Climbing significantly raised only in the EF-p iso group (EF-p iso vs. EF-p: *U* = 5, *p* = 0.004; SF-p iso vs. SF-p: *U* = 16, *p* = 0.09; Figure 5). Again comparisons between EF-p and SF-p groups (Swimming: *U* = 18, *p* = 0.14; Climbing: *U* = 30, *p* = 0.83) and between EF-p iso and SF-p iso groups (Swimming: *U* = 21, *p* = 0.25; Climbing: *U* = 17, *p* = 0.11) were not significant.

Overall, social isolation decreased Immobility and increased Swimming. Moreover, isolated offspring of enriched dams (EF-p iso) exhibited higher levels of Climbing in comparison to their social controls (EF-p).

### c-Fos immunohistochemistry

**Figure 6:**
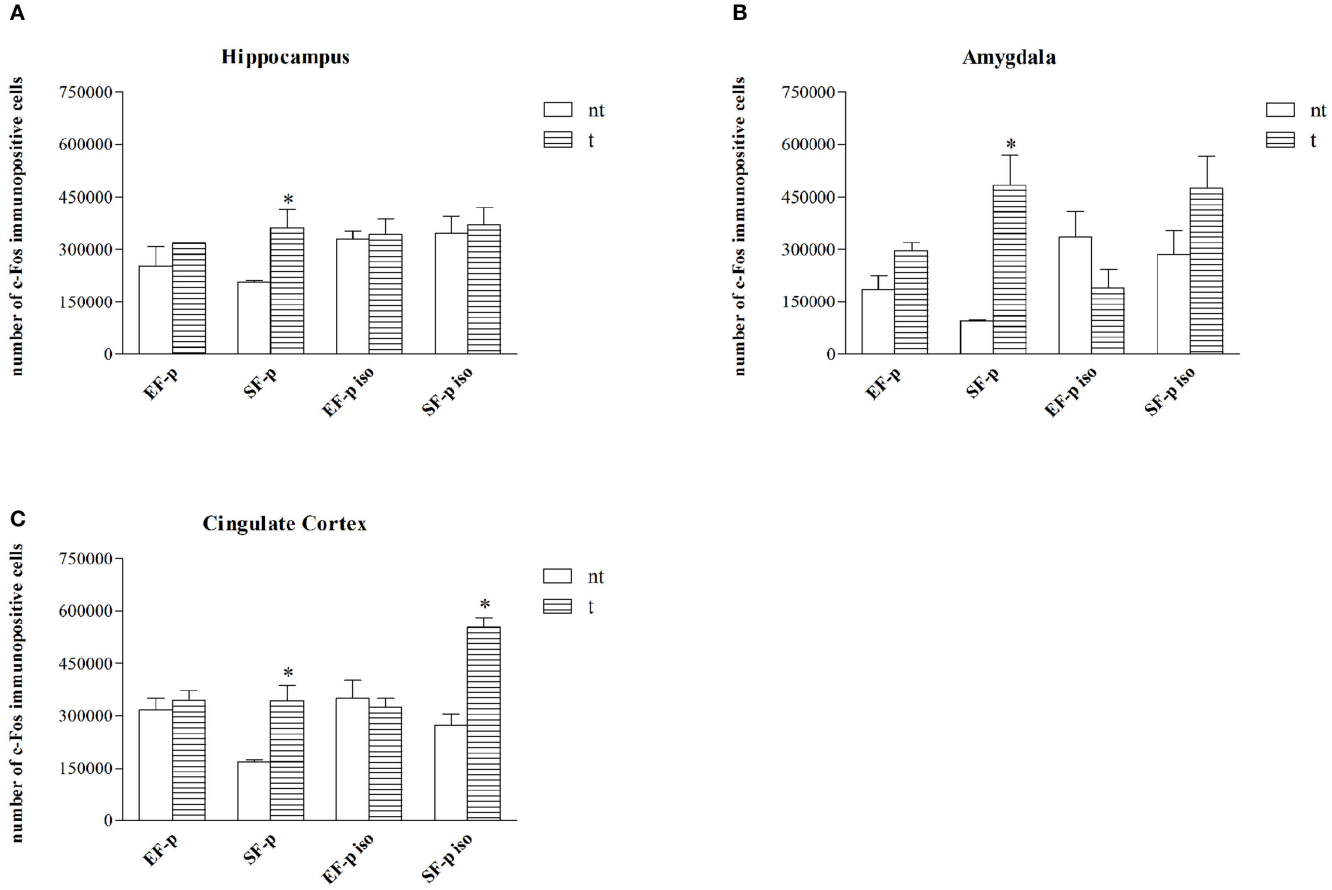
**c-Fos expression**. Histograms show number of c-Fos^+^ cells in: **(A)** Hippocampus, **(B)** Amygdala, **(C)** Cingulate Cortex in EF-p, SF-p, EF-p iso, SF-p iso rats tested (t) or no tested (nt) in FST. ^*^p < 0.05.

In standard reared animals born to standard females (SF-p), the FST induced neuronal activation in all ROI investigated. In fact, SF-p animals tested (*t*) in FST showed a higher number of c-Fos^+^ cells in comparison to no tested (*nt*) SF-p animals in hippocampus, amygdala and cingulated cortex (Hp: *U* = 1, *p* = 0.04, Amyg: *U* = 0, *p* = 0.02; Cg: *U* = 0, *p* = 0.02; Figure 6).

No significant differences were found in all other groups between tested and no tested animals (Table S2), but SF-p iso *t* animals in cingulate cortex (*U* = 0, *p* = 0.02).

**Figure 7:**
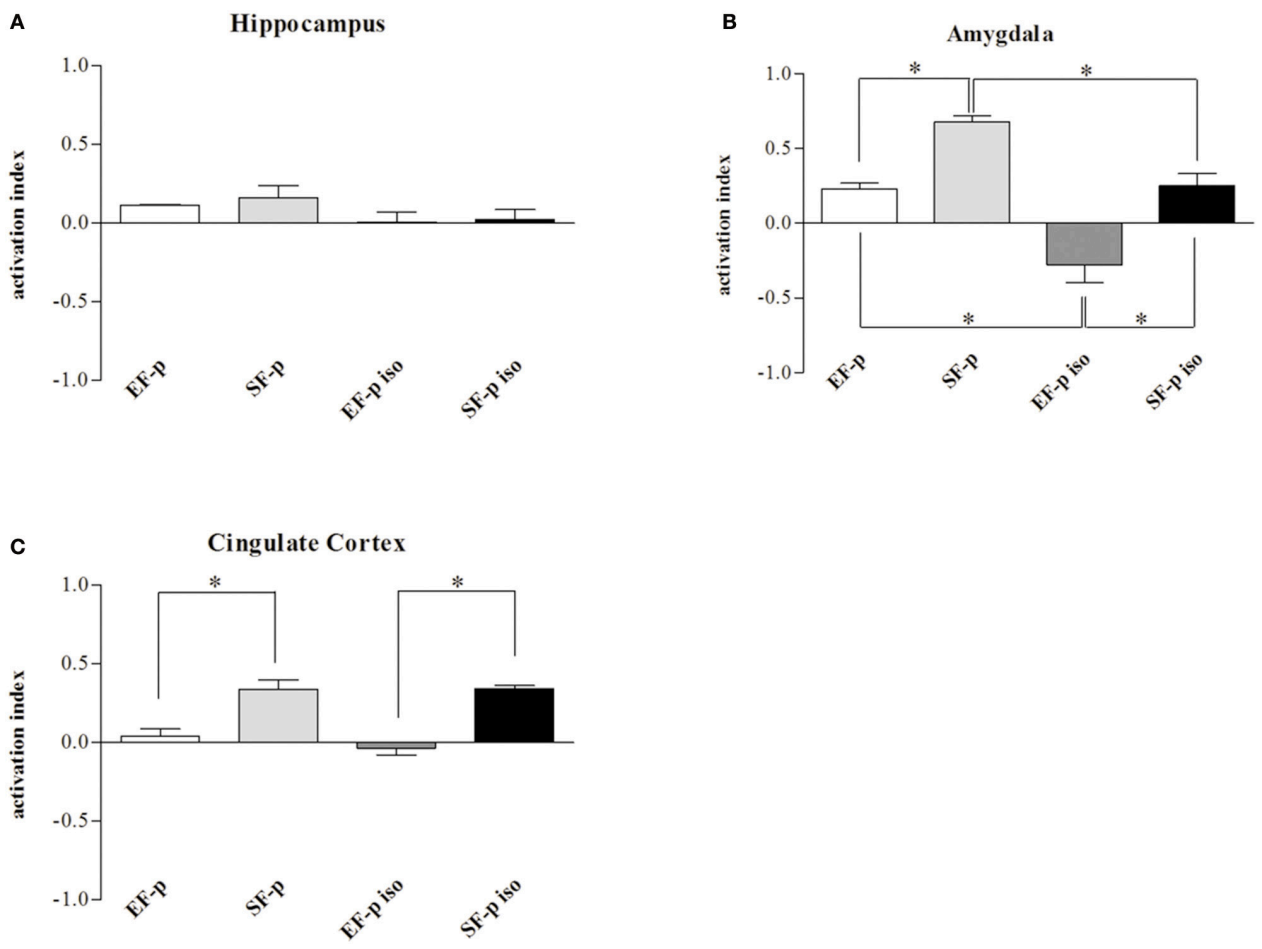
**c-Fos activation index**. Histograms show weighted Δ in **(A)** Hippocampus, **(B)** Amygdala, **(C)** Cingulate Cortex quantified in EF-p, SF-p, EF-p iso, SF-p iso groups. ^*^p < 0.05.

As for c-Fos activation index, no significant differences were found among groups in Hp (*H* = 5.94, *p* = 0.11). Instead, in Amyg (*H* = 12.75, *p* = 0.005) SF-p group showed a significantly higher activation index compared to EF-p (*U* = 0, *p* = 0.02) and SF-p iso (*U* = 0, *p* = 0.02) groups. Furthermore, in Amyg the activation index was higher in EF-p group compared to EF-p iso group (*U* = 0, *p* = 0.02) and lower in EF-p iso group compared to SF-p iso group (*U* = 0, *p* = 0.02). Finally, in Cg (*H* = 11.60, *p* = 0.008) SF-p group showed a higher activation index in comparison to EF-p (*U* = 0, *p* = 0.02) and SF-p iso in comparison to EF-p iso group (*U* = 0, *p* = 0.02; Figure 7).

Overall, the data on neuronal activation revealed a significantly higher number of c-Fos^+^ cells in SF-p rats tested in the FST compared to no tested rats in all areas analyzed. In amygdala both isolation and maternal enrichment reduced the activation index in response to FST, while in Cg only the maternal enrichment reduced it.

### GRs immunohistochemistry

**Figure 8:**
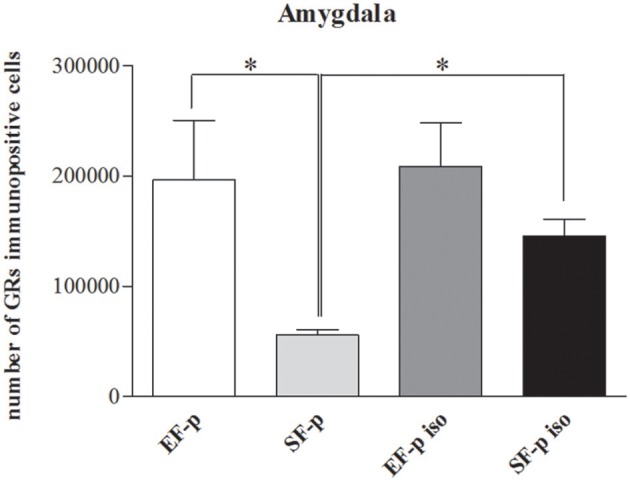
**GRs expression**. Histograms show number of GRs^+^ cells in Amygdala in EF-p, SF-p, EF-p iso, SF-p iso groups. ^*^p < 0.05.

In order to evaluate the effect of maternal rearing condition on the response of their offspring to social isolation, number of GRs^+^ cells was compared among experimental groups. In Amyg (*H* = 9.15, *p* = 0.03) SF-p group showed a significantly lower level of GRs^+^ cells compared to EF-p (*U* = 0, *p* = 0.02) and SF-p iso (*U* = 0, *p* = 0.02) groups. No significant differences were found in the number of GRs^+^ cells of EF-p vs. EF-p iso (*U* = 6, *p* = 0.56) and EF-p iso vs. SF-p iso (*U* = 4, *p* = 0.25). No significant differences among experimental groups were found in all other ROI: Hp (*H* = 5.56, *p* = 0.13) and Cg (*H* = 1.46, *p* = 0.69; Figure 8).

Overall, in not isolated groups the amygdala of enriched dams' offspring exhibited a greater number of GRs in comparison to standard dams' offspring. Social isolation increased GRs levels only in SF-p group.

## Discussion

The present study was designed to evaluate the effects of pre-reproductive maternal experience on maternal behavior and adolescent offspring's response to stress. In line with our previous studies (Caporali et al., 2014, 2015; Cutuli et al., 2015), the pre-reproductive maternal enrichment did influence maternal care and pups' behavior. In fact, in comparison to standard reared females the enriched dams exhibited a more tuned maternal care repertoire, as pointed out by their higher levels of pup-directed behaviors. Pre-reproductive exposure to EE also affected the coping response of the male adolescent offspring when exposed to an inescapable stressful situation (FST) after a chronic stress (social isolation). Namely, after 2 weeks of isolation enriched dams' offspring exhibited higher levels of Climbing during FST in comparison to not isolated offspring born to enriched dams. Furthermore, isolated and control offspring of enriched dams showed a lower amygdaloid c-Fos activation in response to FST and a higher number of GRs compared to offspring of standard reared dams.

Growing evidence indicates that EE influences maternal nurturing. For example, post-weaning enriched housing enhances Licking/Grooming (LG) behavior and oxytocin receptor binding of the low LG offspring across generations (Champagne and Meaney, 2007). Further, EE during pregnancy and lactation enhances pup-licking levels (Sale et al., 2004). Increased levels of pup nursing and licking associated with changes in BDNF and MeCP2 gene expression in the maternal hypothalamus have been also reported in standard reared females mated with an enriched male (Mashoodh et al., 2012). We recently described higher levels of licking, crouching, and nest building activities accompanied by increased BDNF levels in frontal cortex of pre-reproductively enriched dams (Cutuli et al., 2015). Furthermore, in a cross-fostering study we demonstrated that the maternal care of pre-reproductively enriched dams is markedly modulated by the bidirectional interactions between mother and pups, with the enriched dam/standard pups couple resulting in the most maladaptive encounter to shape offspring's phenotype (Caporali et al., 2015). Here, we demonstrate that the early maternal care modifications induced by pre-reproductive EE, which were previously observed using a brief mother-pups separation as eliciting condition (Cutuli et al., 2015), were still evident using undisturbed conditions. In particular, during the observations the enriched dams spent most time in Crouching, the most active and complex nursing posture that allows to nourish and contact the entire litter. Such a behavioral pattern was associated to reduced social investigative (i.e., Sniffing) and partial suckling (i.e., Nursing) behaviors. Concurrently, for the enriched dams the litter assumed a salience able to reduce the emission of non-pup-directed behaviors, such as explorative (i.e., Wall Rearing) or feeding (i.e., Drink or Eat) behaviors.

Generally, the maternal care enhanced by pre-reproductive EE are accompanied by offspring's behavioral and physical improvements. For example, better memory and learning abilities and higher visual acuity have been described in the offspring of enriched mothers compared to the offspring of standard reared dams (Sale et al., 2004; Cutuli et al., 2015). Further, adult male offspring of enriched low LG females display enhanced exploration and novelty discrimination abilities (Champagne and Meaney, 2007). It has also been found that paternal pre-reproductive EE that leads to the above-mentioned maternal care changes is linked to increased weight of their offspring at weaning (Mashoodh et al., 2012). Extending these observations, the present study describes behavioral and biochemical differences in coping response of the isolated adolescent offspring of pre-reproductively enriched females. It is well-known that in adult rats social isolation has a depressant effect that induces increased immobility in FST, while direct exposure to EE has an antidepressant effect that increases swimming and climbing behaviors (Brenes et al., 2008; Mosaferi et al., 2015). In the current research socially isolated adolescent male pups exhibited reduced body weight and paradoxically decreased passive coping responses (Immobility) regardless of maternal pre-reproductive housing. This latter finding indicates that social isolation during early developmental stages alters the response to aversive stimuli at adolescence, in accordance with previous studies (Wongwitdecha and Threenet, 2001; Wongwitdecha et al., 2006; Hong et al., 2012). More importantly, while Swimming was equally increased in both isolated groups (EF-p iso and SF-p iso), maternal enrichment induced higher levels of Climbing during FST in the isolated adolescent offspring (EF-p iso) in comparison to not isolated offspring born to EF (EF-p). Notably, the increased Climbing evident only in EF-p iso suggests that an early chronic stress is required to uncover traits transmitted from mother to pups. In line, Cymerblit-Sabba et al. (2013) found that exposure to EE during pregnancy followed by stress at adulthood improves emotional and attentional reactivity in the offspring.

It is well-known that FST active behaviors reflect the activity of multiple neurotransmitters, with norepinephrine (NE) primarily mediating climbing, and serotonin (5-HT) mediating swimming (Armario et al., 1988; Detke et al., 1995; Rénéric and Lucki, 1998; Cryan et al., 2005). Interestingly, it has been reported that EE enhances NE levels (Naka et al., 2002; Robertson, 2013). Given the increased Climbing in the isolated adolescent rats born to enriched dams, it could be interesting to verify whether the pre-reproductive EE is able to increase NE neurotransmission in the offspring.

Also biochemical analyses support the effects of social isolation and maternal rearing conditions. We found a significantly higher number of c-Fos^+^ cells in SF-p rats tested in the FST compared to no tested SF-p rats in all cortico-limbic structures investigated (Figure 6), endorsing the neuronal activation of structures, such as the hippocampus, amygdala, and cingulate cortex in response to FST (Duncan et al., 1993; Jang et al., 2009; Raineki et al., 2012). Notably, in the remaining groups (SF-p iso, EF-p iso, EF-p) no significant differences between tested and no tested animals were detected, except for SF-p iso group in cingulate cortex (Figure 6C). It is widely recognized that in standard reared animals an acute exposure to a stressful condition (as FST) produces a massive activation of stress response system (Duncan et al., 1993; Jang et al., 2009; Raineki et al., 2012) that can be altered by a persistent exposure to a stressful condition (as social isolation; Chen and Herbert, 1995; Wall et al., 2012). Also EE is able to induce physiological adaptations of stress system by decreasing neuronal activation (Solinas et al., 2009; Grimm et al., 2016). The present results extend the reduced activation of stress response even to animals not directly exposed to an enriched environment. Namely, the neuronal activation of amygdala was influenced by both social isolation and maternal EE in a summative way (Figure 7B). The findings on the amygdala exemplify how an early chronic stress uncovers the intergenerational EE effects on the response to an inescapable stress. Several studies have shown that enhancements of amygdaloid responses can be associated to develop psychopathology (LeDoux, 2000; Davis et al., 2010; Rajbhandari et al., 2016; Rincón-Cortés and Sullivan, 2016; Shackman et al., 2017). Among others, Raineki et al. (2012) demonstrated that an early-life abuse is followed by depressive-like behaviors at adolescence, which are related to amygdaloid hyperactivation and rescued by temporary pharmacological deactivation of the amygdala. Consistently, we found that EF-p iso group showed the lowest c-Fos activation index, demonstrating that a post-weaning isolation is able to reduce amygdaloid activation and that the being born to a pre-reproductive enriched female further blunts c-Fos expression.

The amygdala with its projections to the paraventricular hypothalamic nucleus, bed nucleus of the stria terminalis and periaqueductal gray (Allen and Allen, 1974; Van de Kar et al., 1991; Herman et al., 2005) is markedly involved in stress response circuitry and further clue of this modulation raises from its GRs expression. GRs are recognized as a great marker of brain plasticity, adaptation, and vulnerability (de Kloet et al., 2008). Genomic and non-genomic GRs signal transductions have considerable consequences for the individual's response and adaptation to stressors. Thus, a flourishing field of literature is rooted on corticosteroid receptor role with the goal to explain how stress can lead to multifaceted outcomes, ranging from vulnerability to stress resilience (Meaney et al., 1985; de Kloet et al., 2005; Schulte-Herbrüggen et al., 2006; Oitzl et al., 2010). We found significant differences in amygdala GRs expression associated with both offspring's social isolation and pre-reproductive rearing conditions of dams, as demonstrated by the higher amygdaloid GRs expression in SF-p iso and EF-p groups compared to SF-p group (Figure 8). Noteworthy, no significant differences were found between EF-p and EF-p iso groups, suggesting that the higher amygdaloid GRs expression of EF offspring could have been transmitted from mother to pups and no further modulated by social isolation.

Models of genetically altered animals have provided a useful tool to investigate GRs role in the stress response. Models of murine depression and stress-resistance have been generated by using the GR-heterozygous mutant mice (GR^+/−^) that under-express GRs, or the YGR mice that over-express GRs (Ridder et al., 2005; Schulte-Herbrüggen et al., 2006). Namely, GR overexpression-dependent increases of BDNF in hippocampus and amygdala appear to be the dynamic correlate of enhanced stress resistance (Schulte-Herbrüggen et al., 2006). Since the transmission of positive (Caporali et al., 2014; Cutuli et al., 2015) and negative (Niknazar et al., 2016) maternal experiences affects offspring's BDNF levels, it is possible that the greater resilience of the EF-p iso group over-expressing amygdaloid GRs may be ascribed to enhanced BDNF levels.

As final note, we would like to emphasize that EF offspring's behavioral performance likely occurs through alterations in maternal care due to the pre-reproductive EE (Champagne and Meaney, 2007). In fact, the increase in Crouching levels observed in enriched dams make them similar to the high Licking and Grooming and Arched-Back Nursing (LG-ABN) mothers described by Meaney's group (Weaver et al., 2004), and it may account for the increase in Climbing exhibited by their offspring in facing the FST. Similarly, the offspring of high LG-ABN mothers exhibited enhanced exploration and reduced anxiety accompanied by attenuated HPA response to stress and an over-expression of GRs, although in the hippocampus (Liu et al., 1997, 2000; Caldji et al., 1998; Champagne and Meaney, 2007).

We are aware that the intergenerational transmission of EE effects might also occur through maternal germ-line, during fetal development, and lactation (Franklin and Mansuy, 2010; Ho and Burggren, 2010), but this topic is out of the current study scope. Anyway, the outcome of the perinatal interaction “gene × environment” depends on the degree of “matching” between intergenerationally transmitted features and environmental demands later in life. Not by chance, the main behavioral and biochemical differences observed in EF-p group become indeed evident when the pups are socially isolated.

## Conclusions

After the beneficial effects on motor and cognitive abilities (Caporali et al., 2014; Cutuli et al., 2015), we now show the influence of pre-reproductive maternal EE on offspring's stress response.

Notably, offspring of parents exposed to negative experiences (stress or trauma) have been reported to be at a greater risk for physical, behavioral, and cognitive problems (Yehuda and Bierer, 2008; Roth et al., 2009; Debiec and Sullivan, 2014; Bowers and Yehuda, 2016). On the other hand, positive complex stimulation, such as EE, seems to intergenerationally produce a neuroplastic reserve able to counteract negative outcomes of stressful events through multiple mechanisms, such as GRs modulation. Thus, evaluating whether this reserve can be spent beyond adolescence to cope with stress during the entire lifespan is an important issue deserving investigations in the near future. In the same manner, it could be interesting to assess whether maternal care modifications induced by the pre-reproductive EE are stable in time and across generations. Further, since in the present research the amygdala has proved to be the brain area most sensitive to stress and intergenerational EE influence, the implication of amygdaloid circuits in emotional regulation should be thoroughly investigated using the paradigm of pre-reproductive EE.

## Author contributions

All authors participated in designing the research; DC, PC, PS, DL, and FF performed behavioral evaluations; PD, EB, GP, MP, and FG performed biochemical analyses; DC, PC, PD, EB, GP, MP, and LP analyzed data; all authors discussed and approved data; DC, EB, GP, MP, DL, and LP interpreted data and wrote the paper.

### Conflict of interest statement

The authors declare that the research was conducted in the absence of any commercial or financial relationships that could be construed as a potential conflict of interest.
