# Supplementary material for: Influence of Pre-reproductive Maternal Enrichment on Coping Response to Stress and Expression of c-Fos and Glucocorticoid Receptors in Adolescent Offspring
Source: Front Behav Neurosci. 2017 May 9;11:73. doi: 10.3389/fnbeh.2017.00073 (PMC5422443; doi:10.3389/fnbeh.2017.00073)
Supplement: Supplementary file 1 [file Table1.DOCX]

**Table 1S.** Results of Mann-Whitney U tests performed on maternal behavior of EF and SF dams. Table shows U and p value of comparisons between groups in each behavioral parameter.

| **A. *pup-directed behaviors*** | | **B. *non pup-directed behaviors*** | |
| --- | --- | --- | --- |
| **Sum**  Duration  Frequency | U = 12, p = **0.04** **(EF↑)**  U = 18, p = 0.14 | **Sum**  Duration  Frequency | U = 8, p = **0.01** **(EF**↓**)**  U = 14, p = 0.06 (EF↓) |
| **Retrieving**  Duration  Frequency | U = 29, p = 0.75  U = 28.5, p = 0.70 | **digging**  Duration  Frequency | U = 24.5, p = 0.43  U = 26, p = 0.53 |
| **Licking**  Duration  Frequency | U = 24, p = 0.40  U = 26, p = 0.53 | **Grooming**  Duration  Frequency | U = 20, p = 0.21  U = 18.5, p = 0.15 |
| **Sniffing**  Duration  Frequency | U = 4, p = **0.003** **(EF↓)**  U = 4, p = **0.003** **(EF↓)** | **Wall Rearing**  Duration  Frequency | U = 14, p = 0.06 (EF↓)  U = 17, p = 0.11 |
| **Nursing**  Duration  Frequency | U = 0, p = **0.0008 (EF↓)**  U = 0, p = **0.0007 (EF↓)** | **Exploring**  Duration  Frequency | U = 19, p = 0.17  U = 14.5, p = 0.07 |
| **Crouching**  Duration  Frequency | U = 0, p = **0.0008** **(EF↑)**  U = 8, p = **0.01** **(EF↑)** | **Resting**  Duration  Frequency | U = 20, p = 0.21  U = 16, p = 0.09 |
| **Nest Building**  Duration  Frequency | U = 27.5, p = 0.63  U = 29.5, p = 0.79 | **drink or eat**  Duration  Frequency | U = 10, p = **0.02** (EF↓)  U = 7, p = **0.01** (EF↓) |
| **C. *other behaviors*** | | | |
| Duration  Frequency | | U = 32, p = 1  U = 26, p = 0.53 | |
