# Supplementary material for: Influence of Pre-reproductive Maternal Enrichment on Coping Response to Stress and Expression of c-Fos and Glucocorticoid Receptors in Adolescent Offspring
Source: Front Behav Neurosci. 2017 May 9;11:73. doi: 10.3389/fnbeh.2017.00073 (PMC5422443; doi:10.3389/fnbeh.2017.00073)
Supplement: Supplementary file 2 [file Table2.docx]

**Table 2S.** Non-significant results of Mann-Whitney U tests: EF-p, SF-p, EF-p iso, SF-p iso in ROI (Hippocampus, Hp; Amygdala, Amyg; Cingulate Cortex, Cg). Table shows U and p value of non-significant comparisons between **t**/**nt** in each group.

|  |  |  |  |
| --- | --- | --- | --- |
| **ROI** | **Group** | **U** | **p** |
| Hp | EF-p | 4 | 0.24 |
|  | EF-p iso | 7 | 0.77 |
|  | SF-p iso | 5 | 0.39 |
| Amyg | EF-p | 3 | 0.15 |
|  | EF-p iso | 3 | 0.15 |
|  | SF-p iso | 2 | 0.08 |
| Cg | EF-p | 5 | 0.38 |
|  | EF-p iso | 8 | 1 |
